# High prevalence of fecal carriage of extended-spectrum β-lactamase-producing Escherichia coli and Klebsiella pneumoniae in a pediatric unit in Madagascar

**DOI:** 10.1186/1471-2334-10-204

**Published:** 2010-07-12

**Authors:** Todisoa Andriatahina, Frédérique Randrianirina, Eliosa Ratsima Hariniana, Antoine Talarmin, Honoré Raobijaona, Yves Buisson, Vincent Richard

**Affiliations:** 1 Hôpital Joseph Raseta Befelatanana, Antananarivo, Madagascar; 2 Institut Pasteur, Antananarivo, Madagascar; 3 Institut de la Francophonie pour la Médecine Tropicale, Ventiane, Laos

## Abstract

**Background:**

Extended-spectrum β-lactamase (ESBL)-producing *Enterobacteriaceae *have spread worldwide but there are few reports on carriage in hospitals in low-income countries. ESBL-producing *Enterobacteriaceae *(ESBL-PE) have been increasingly isolated from nosocomial infections in Antananarivo, Madagascar.

**Methods:**

we conducted a prevalence survey in a pediatric unit from March to April 2008 Patient rectal swabs were sampled on the first and the last day of hospitalization. Medical staff and environment were also sampled. Rectal and environmental swabs were immediately plated onto Drigalski agar supplemented with 3 mg/liter of ceftriaxon.

**Results:**

Fecal carriage was detected in 21.2% of 244 infants on admission and 57.1% of 154 on discharge, after more than 48 hours of hospitalization (p < 0.001). The species most frequently detected on admission were *Escherichia coli and Klebsiella pneumoniae *(36.9%), whereas, on discharge, *K. pneumoniae *was the species most frequently detected (52.7%). ESBL-associated resistances were related to trimethoprim-sulfamethoxazole (91.3%), gentamicin (76.1%), ciprofloxacin (50.0%), but not to amikacin and imipenem. The increased prevalence of carriage during hospitalization was related to standard antimicrobial therapy.

**Conclusion:**

The significant emergence of multidrug-resistant enteric pathogens in Malagasy hospitals poses a serious health threat requiring the implementation of surveillance and control measures for nosocomial infections.

## Background

New classes of enzymes conferring resistance to β-lactam antibiotics have emerged over the last few decades, due to antibiotic selection pressure; most alarming are the extended spectrum β-lactamases (ESBLs) produced by enteric pathogens that have spread worldwide since their first description in 1983 [1]. Typically, ESBLs hydrolyze third generation cephalosporins and aztreonam, but not carbapenems, and are inhibited by clavulanic acid and tazobactam [2]. ESBL-producing pathogens frequently exhibit plasmid-encoded multidrug resistance. Therefore, antibiotic therapy for treating these infections is limited to a small number of expensive drugs. As a result of mutations, more than 200 types of ESBLs are currently described in various species of the *Enterobacteriaceae *family and other non enteric organisms, such as *Pseudomonas aeruginosa *and *Acinetobacter sp*. TEM- and SHV-type β-lactamases, mainly produced by *Klebsiella pneumoniae*, have spread throughout hospital settings, and CTX-M enzymes, mainly produced by *Escherichia coli*, have become predominant in the community [3,4].

Since the 1990s, nosocomial outbreaks due to ESBL-producing *Enterobacteriaceae *(ESBL-PE) have been increasingly reported worldwide, especially in developed countries [5].

In hospital settings, intestinal carriage is the main reservoir of these organisms. The gut colonization of inpatients is associated with a high risk for developing self and cross infections due to ESBL-producers, especially in long-term care units. Dissemination of ESBL-producing clones results from the movement of patients between various units of the same hospital, but also between hospitals, nationally or internationally [6,7]. In low-income countries, prevalence studies on the carriage of ESBL-producing Gram negative bacilli (GNB) are scarce, whereas the burden of linked infections is increasing due to an absence of expensive second-line antibiotics [8]. A facility-based study on neonatal sepsis in India showed that 50% of causative GNB were ESBL-PE [9]. Few studies have evaluated fecal carriage during non outbreak situations. Patients colonized at admission can nevertheless introduce the pathogen into hospital units [4].

ESBL-PEs in Madagascar were initially isolated between 2005 and 2006 from community-acquired urinary tract infections [10]. They were then isolated during an epidemic that occurred in two pediatric units in 2006; more recently, ESBL-PEs have been isolated from several infections acquired in various surgical and intensive care units of Antananarivo [11].

This study, conducted in a pediatric department of a large teaching hospital in Antananarivo, aimed to assess the levels of ESBL-PE carriage among hospitalized infants -- on admission and on discharge -- and to determine the risk factors for colonization and infection.

## Methods

### Design of the study

Patients <15 years of age hospitalized in the pediatric unit of Befelatanana hospital in Antananarivo were enrolled, after receiving parental consent, in a cohort study from March to April 2008. The unit has a capacity of 22 rooms and 105 beds managed by a staff of 15 physicians, 22 nurses and 15 employees. For each patient, the demographic characteristics (age, gender...), medical history (prior hospitalization, prior invasive devices used...), antibiotic therapies, diagnosis on admission and discharge were recorded. Patient rectal swabs were sampled on the first and the last day of hospitalization. Rectal swabs were also sampled among voluntary medical staff during the same period. Moreover, each week, 20 environmental sites (table, sink, stethoscopes...) were sampled in the four care rooms of the unit.

### Laboratory methods

Rectal and environmental swabs were immediately plated onto Drigalski agar supplemented with 3 mg/liter of ceftriaxone. The plates were forwarded to the Institut Pasteur of Madagascar within 4 hours for 24- to 48-hour incubation at 37°C. All GNB isolates were identified by Gram staining, and by API 20E and API 20NE system (bioMérieux, Marcy l'Etoile, France). The isolates were then screened for ESBL production using both the resistance phenotype and the double-disk synergy test using conventional combination: cefotaxime, ceftazidime, ceftriaxone and amoxicillin-clavulanic acid [2,12]. The organisms were considered to be producing ESBL when the zone of inhibition around any of the expanded-spectrum cephalosporin discs showed a clear-cut increase towards the clavulanic acid disc. For *E cloacae *strains, this was confirmed by the double disc potentiation test using a cefepime and clavulanic disc. Phenotypic disc confirmatory test was performed as recommended by the Clinical and Laboratory Standards Institute (CLSI -2005).

Antibiotic susceptibility was tested by the standard disk diffusion method with OSIRIS system (Biorad, Marne la Coquette, France) on Mueller-Hinton agar, as recommended by the Antibiogram Committee of the French Microbiology Society (ACFMS) for the characterization of ESBLs [3]. Amoxicillin, ticarcillin, gentamicin, tobramycin, amikacin, imipenem, nalidixic acid, ciprofloxacin, cotrimoxazole were also tested to determine the resistance patterns of the isolates.

### Statistical analysis

Data entry and analysis were performed with Statistica Software, version 5.5 (Statsoft Corporation, OK, USA). The Chi-square test and Fisher's exact test were used for univariate analysis, with the ANOVA and Kruskall Wallis tests used for comparison of medians. P-values <0.05 were considered to be statistically significant. Explanatory variables associated with a p-value less than 0.20 were analyzed by logistic regression to investigate the confounding factors.

### Ethical clearance

The study was approved by the Ministry of Health and the National Ethics Committee of Madagascar. Informed consent was obtained from at least one parent of each child hospitalized before enrolment.

## Results

### Hospital characteristics with respect to patients

#### Prevalence of ESBL-PE carriage

##### Patients

Thirty-seven patients were already hospitalized at the start of the study. For these patients, the sex ratio (male/female) was 1.1 and the mean age was 53.1 months (95%CI: 33.3-73.0). Twenty patients were infected with ESBL-PE (54.1%), particularly *K. pneumoniae *(n = 11) and *E. coli *(n = 8).

From March 10 to April 11, 281 patients were admitted to the pediatric unit. Consent was not obtained for 22 patients (7.8%). Overall, of a possible 259 patients, 15 patients (5.8%) were not included, as they were admitted to the hospital on the last day of the prospective study.

Of the 244 patients that were included in the cohort, 90 (36.8%) could not be sampled on discharge for various reasons: 17 (18.8%) died during hospitalization, 19 (21.1%) left the unit without medical advice and 54 (60.0%) were hospitalized for less than 48 h. Rectal swabs were collected for 154 (63.1%) patients on discharge.

**Figure 1:**
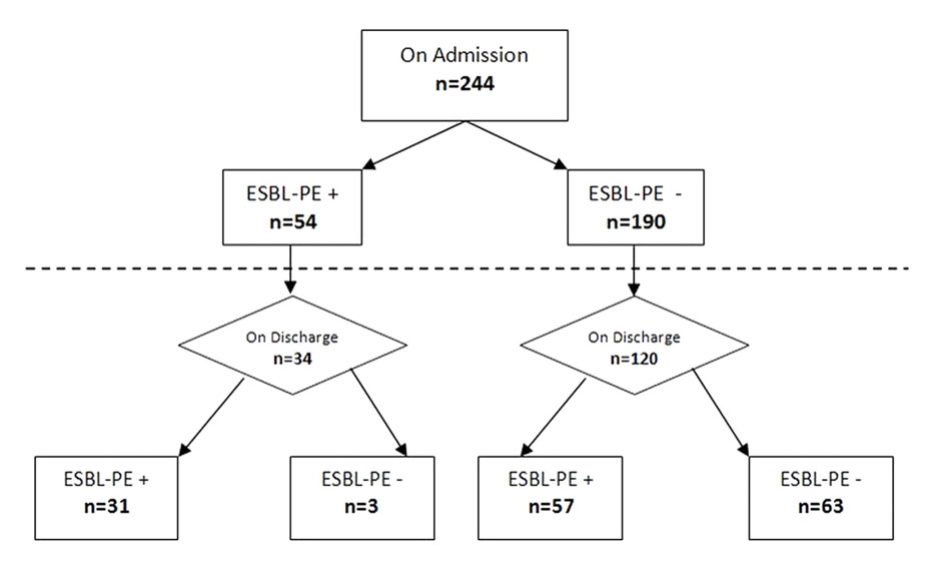
**Included patients distribution according to ESBL-PE carriage status on admission and on discharge**.

Cohort (244 patients) characteristics included a sex ratio of 1.25, a mean age of 38.3 months (95%CI: 83.1-95.5). For all the patients included and discharged before the end of the study, the mean length of hospital stay was 5.7 days (95% CI: 5.2-6.2). Main admitting diagnoses included respiratory (31.5%), infectious (20.9%), digestive (12.3%) and nutritional disorders (15.2%). On admission, none diagnosed infection was in relation with ESBL-producing microorganism. A total of 54/244 patients (22.1%) were detected as ESBL-PE carriers on admission, and 88/154 (57.1%) were ESBL-PE carriers on discharge (p-value for trend <0.001). Among the ESBLE-PE carriers on admission (n = 54), 34 could be sampled on discharge. Among the 31 that were still ESBL-PE carriers; 12 (38.7%) harbored a different organism. On discharge among the 120 sampled patients who were not carriers on admission, 57 (47.5%) were detected as ESBL-PE carriers (Figure 1).

Carriage rates on admission and discharge did not differ according to the age groups (table 1), but the relative risk of hospital acquisition was significantly higher in the 5-9 year-old group (RR = 6.1 [95%CI: 1.9-19.4]).

**Table 1:** ESBL carriage rate on admission and discharge as a function of age group

Age group	On admission		On discharge		RR [95%CI]
	**N**	**ESBL + (%)**	**N**	**ESBL + (%)**	

0-4 years	182	44 (24.2)	115	70 (60.8)	2.5 [1.9 - 3.5]
5-9 years	35	3 (8.5)	21	11 (52.4)	6.1 [1.9 - 19.4]
≥10 years	27	7 (25.9)	18	7 (38.8)	1.5 [0.7 - 3.5]
Total	244	54 (22.1)	154	88 (57.1)	2.6 [2.0 - 3.5]

##### Medical Staff

Among the medical staff (Table 2), 39 of 52 individuals (75.0%) agreed to participate in the study. ESBL-PE, *E. coli *(n = 9) and *K. pneumoniae *(n = 9), were detected in the rectal swab cultures from 19 medical staff (48.7%). Carriage rates did not differ according to the period of the study.

**Table 2:** ESBL-producing Enterobacteria according to the different origins of the samples

	Samples taken	Positive samples N, (%)	ESBL-producing strains	*Escherichia coli* N, (%)	*Klebsiella Pneumoniae* N, (%)	*Enterobacter cloacae* N, (%)	Other enterobacteria N, (%)
Patient hospitalized at the beginning of the study	37	20 (54.1)	20	8 (40.0)	11 (55.0)	1 (5.0)	0
Patients on admission	244	54 (22.1)	65	24 (36.9)	24 (36.9)	11 (16.9)	6 (9.2)
Patients on discharge	154	88 (57.1)	110	30 (27.3)	58 (52.7)	11 (10.0)	11 (10.0)
Medical Staff	39	19 (48.7)	20	9 (45.0)	9 (45.0)	1 (5.0)	1 (5.0)
Environmental samples	100	13 (13.0)	13	4 (30.8)	5 (38.5)	4 (30.8)	0
Nosocomial infections	7	6 (85.7)	5	0	3 (60.0)	0	2 (40.0)
**Total**	**578**	**200 (34.6)**	**233**	**75 (32.2)**	**110 (47.2)**	**28 (12.0)**	**20 (8.6)**

##### Environmental sample

At least one isolate of ESBL-PE was detected in each room sampled during the study. Of the 13 positive samples (Table 2), the main species were *K. pneumoniae *(n = 5), *Enterobacter cloacae *(n = 4) and *E. coli *(n = 4). *K. pneumonia *were detected in each care room, *E.Coli *and *E. Cloacae *in three of the four care rooms. No ESBL-PE isolate was detected during the second week of the study.

### Characteristics of ESBL-PE isolates

At all 233 ESBL-PE isolates were found: *K. pneumoniae *(47.2%), *E. coli *(32.2%) *E. cloacae *(12.0%) and other enterobacteriaceae (8.6%) (Table 2).

On admission, *E. coli *and *K. pneumoniae *was the most frequent ESBL-PE identified (36.9%, 24/65), whereas *K. pneumoniae *was the predominant species on discharge (52.7%, 58/110), in patients already hospitalized at the start of the study (55%, 11/20), in medical staff (45.0%, 9/20), in environment (38.5%, 5/13) and in nosocomial infection (60.0%, 3/5) (Table 2).

All isolates showed similar resistance patterns. All were susceptible to imipenem and amikacin with cross-resistance to trimethoprim-sulfamethoxazole (91.3%), gentamicin (76.1%) and ciprofloxacin (50.0%). *K. pneumoniae *isolates were significantly less resistant to ciprofloxacin than *E. coli *isolates (34.5% vs 68.4%; p-value < 0.001).

Community and hospital acquired strains had the same resistance profile (Table 3).

**Table 3:** Antimicrobial resistance of ESBL-producing *E. Coli *and *K. Pneumoniae *isolates from community or acquired during hospitalization (number of tested isolates in brackets)

	Drugs											
		AMX	TIC	AMC	CAZ	GEN	TOB	AMK	IMP	NAL	CIP	TMP
***Community strains - On admission without prior hospitalization history***												
*E. coli (n = 15)*	%	100.0	100.0	100.0	93.3	73.3	80.0	0.0	0.0	86.7	60.0	93.3
*K.Pneumoniae (n = 15)*	%	100.0	100.0	100.0	86.7	80.0	86.7	0.0	0.0	40.0	33.3	93.3
												
***Hospital-acquired strains - On discharge ESBL-PE negative on admission***												
*E. coli (n = 19)*	%	100.0	100.0	100.0	100.0	94.7	100.0	0.0	0.0	94.7	78.9	89.5
*K.Pneumoniae (n= 39)*	%	100.0	100.0	100.0	79.4	89.7	89.7	0.0	0.0	46.1	35.8	100.0

AMX:Amoxicillin, TIC:Ticarcillin, AMC: Amoxicillin+Clavulanic Acid, CAZ: Ceftazidime, GEN: Gentamicin, TOB:Tobramycin, AMK: Amikacin, IMP: Imipenem, NAL: Nalidixic Acid, CIP: Ciprofloxacin, TMP: Trimethoprim/Sulfamethoxazole.

### Risk factors for ESBL-PE carriage

In univariate analysis, significant risk factors for ESBL-PE carriage on admission were prior hospitalization, use of invasive devices in the last 30 days, and infection upon admission (Table 4). In multivariate analysis, four variables were first included in the model: Patient origin, prior hospitalization, use of invasive devices in the last 30 days, infection upon admission. Using backward stepwise procedure, prior hospitalization in the last 30 days (adjusted OR = 7.4 [95%CI: 2.9-18.3]) was the only independent risk factor for ESBL-PE carriage.

**Table 4:** Analysis of the risk factors on admission

	Total (n = 244)		ESBL + (n = 54)			
	N	(%)	N	(%)	OR	p-value
**Sex**						
Male	136	(55.7)	32	(59.2)	1.3	0.5
**Age group (months)**						
<1	31	(12.7)	6	(11.1)		0.31
1 to 12	84	(34.4)	25	(46.3)		
13 to 24	37	(15.2)	8	(14.8)		
25 to 35	22	(9.0)	4	(7.5)		
≥36	70	(28.7)	11	(20.3)		
**Mean age **[95%CI]	35.6	[29.7-41.6]	33.3	[19.2-47.4]		0.86
						
**Patient origin**						
House	73	(29.9)	15	(27.8)		0.2
Primary Health centre	149	(61.0)	31	(57.4)		
Other unit or hospital	22	(9.0)	8	(14.8)		
**Last 30 days history**						
Hospitalization	23	(9.4)	14	(25.9)	7.4	<0.01
Invasives devices used	15	(6.1)	11	(20.3)	10.8	<0.01
Antibiotic therapy	100	(40.9)	22	(40.7)	1.0	0.9
**Admitting diagnoses**						
Respiratory	84	(31.5)	19	(35.2)	0.8	0.5
Digestive	39	(12.3)	9	(16.6)		
Malnutrition	37	(15.2)	10	(18.5)		
Neurological	49	(20.1)	8	(14.8)		
						
**Infection upon admission**	51	(20.9)	19	(37.3)	1.3	<0.01
						

Significant risk factors for ESBL-PE hospital acquisition in univariate analysis were antibiotic therapy and intramuscular injections (Table 5). Ages of patients did not differ between ESBL-PE carriers (mean: 34.2 months) and non carriers (mean: 39.6 months). In multivariate analysis, five variables were first included in the model: antibiotic therapy, intracranial catheter, brachial catheter, intramuscular injections and care rooms used. After backward stepwise procedure, antibiotic therapy (adjusted OR = 4.1 [95%CI: 1.8-9.4]) was the only independent risk factor for ESBL-PE acquisition.

**Table 5:** Analysis of the risk factors during hospitalization

	Total (n = 120)		ESBL-PE Carriage (n = 57)				
	n	(%)	n	(%)	OR	95%CI	p-value
**Hospitalization treatments**							
Antibiotics	77	(64.2)	45	(78.9)	3.5	[1.5-5.9]	<0.01
Intracranial catheter	38	(31.7)	22	(38.6)	1.9	[0.9-4.4]	0.08
Brachial catheter	20	(16.7)	13	(22.8)	2.3	[0.8-6.4]	0.1
intramuscular injection	36	(30.0)	24	(42.1)	3.1	[1.4-7.2]	<0.01
intravenous injection	10	(8.3)	4	(7.0)	0.7	[0.2-2.7]	0.5
Nasogastric sond	30	(25.0)	16	(28.1)	1.1	[0.7-1.7]	0.7
							
**Moving in unit**							
rooms	35	(29.2)	17	(29.8)	0.95	[0.6-1.4]	0.7
care rooms	107	(89.2)	54	(94.7)	1.01	[0.6-1.8]	0.9
							
**Care rooms used**							
Ground floor	7	(5.8)	6	(10.5)	3.63	[0.6-22.4]	0.1
First floor	40	(33.3)	18	(31.6)	1.4	[0.4-5.5]	0.5
Second floor	60	(50.0)	30	(31.6)	1.3	[0.3-5.9]	0.9
							

During hospitalization, 64.2% of the patients were treated by antibiotic therapy. The average number of antibiotics prescribed by patient was 1.2 (95%CI: 1.0-1.4). For patients with ESBL-PE carriage on discharge the average number of antibiotics prescribed was significantly higher than in patients with no-ESBL-PE carriage (1.7 vs 0.9; p-value < 0.01). Overall, 46.8% of patients received more than one antibiotic. The antibiotic therapy included ampicillin (38.7%), ceftriaxon (29.4%) and gentamicin (27.8%). In backward stepwise logistic regression analysis, ampicillin (adjusted OR = 3.9 [95%CI: 1.6-9.9]) was the only antibiotic in relation with ESBL-PE acquisition (table 5).

### Nosocomial infections

During the study, six hospital-acquired infections occurred among the 190 patients hospitalized for more than 48 hours. The incidence rate was estimated at 3.1% during the study period. The major cause of infection was intravenous catheters (n = 4). None of the six patients were ESBL-PE carriers on admission.

*K. pneumoniae *(n = 3), *Pantoea sp *(n = 2), *Acinetobacter baumanii *(n = 1) were isolated. All *K. pneumoniae *and *Pantoea sp *were ESBL-PE but remained susceptible to amikacin and imipenem in all cases.

## Discussion

This study aimed to evaluate the intestinal carriage of ESBL-PE in children hospitalized in the JRB Hospital of Antananarivo, to develop a preventive strategy for controlling the spread of multiresistant bacteria in the pediatric ward. Our results confirm previous findings showing high levels of gut colonization by ESBL-PE in hospitals of low-income countries [3], and raises special concerns about the community spread of these multidrug resistant bacteria in Madagascar.

On admission, a carriage rate greater than 20% suggests prior acquisition via the community. Interestingly, although history of hospitalization appeared to be a risk factor, 74.1% of ESBL-PE carriers had not been hospitalized during the previous 30 days (Table 4), demonstrating that the community compartment is essential for the maintenance of ESBL-PE. Of the 154 patients sampled on discharge after more than 48 hours of hospitalization, the carriage rate exceeded 50%. The independent risk factors associated with ESBL-PE carriage were however known. Thus, previously hospitalized patients receiving antibiotic therapy, especially ampicillin, are the patients most likely to be affected. Additional risk results from placement of indwelling devices (catheters). Moreover, 48.7% healthy medical staffs were carriers, and, 22% of the environmental samples were positive, indicating poor hospital hygiene.

Swabs were plated on Drigalski agar supplemented with 3 mg/liter ceftriaxon; this led to the selection of not only ESBL-PE, but also to all GNB resistant to third generation cephalosporins, including non-fermentative species as *P. aeruginosa *or *Acinetobacter baumannii*. Although the recommended selective media is Drigalski agar supplemented with cefotaxime 0.5 mg/liter or MacConkey agar supplemented with ceftazidime 4 mg/liter, ceftriaxone was chosen, as it is the only third generation cephalosporin available in Madagascar. The concentration of 3 mg/liter was adjusted by pretesting, to improve specificity of the screening. It is therefore likely that the true prevalence rates of ESBL-PE carriage were higher than those found, already well above the rates reported elsewhere. Conversely, studies conducted from 1993 to 1997 in Finland [13] or from 1997 to 1999 in Spain [14] showed that stool cultures contained no ESBL-producing isolates when performed on antibiotic-free agar plates.

A weakness of the study was that we were unable to sample all of the patients on discharge, diminishing the strength of the statistical analysis. However, the rate of 50% for ESBL-PE carriage on discharge, in a non epidemic context, matched well that of inpatients sampled at the beginning of the study.

Asymptomatic colonization of the intestinal mucosa with ESBL-producing GNB has been previously described [15-17]. Several surveys, conducted during nosocomial infection outbreaks, revealed the spread of multiresistant strains within the ward, including the spread to other wards of the hospital due to transmission from patients to others or from staff to patients. In our study, we were not able to determine the relatedness between ESBL-PE from different origins, but the origins are probably various. Rates of ESBL-PE carriage among hospitalized patients were estimated at 11.7% in Spain [18], 16% in Lebanon [19], and 26% in Saudi Arabia [20]. A previous prospective study of children in Turkey revealed that 18.5% of children carry ESBL-producing *K. pneumoniae*, the incidence rate of nosocomial infections due to this strain being 1.6% among hospitalized children [21].

The rate of 22.1% of ESBL-PE carriage on admission to the pediatric ward in Antananarivo, largely exceeds that reported from an intensive care unit in a hospital in Baltimore (2%) [22], and from a neonatal intensive care unit in Washington (4.2%) [23]. All these findings and those of outpatients in Barcelona, Spain (7.5%) [4] raise the question of asymptomatic carriage of ESBL-PE in the community [7], which enhances the spread of resistance genes by human-to-human transmission or contamination of the environment [24,25]. Such a hidden reserve of resistance factors carried by healthy people may help to maintain high levels of antimicrobial resistance in hospital floras [26,27]. Under antibiotic selective pressure, especially in intensive care units [28], intestinal colonization by ESBL-PE strains is favored among inpatients, increasing the carriage rates and the risk of nosocomial infections [29,30]. First-line antibiotic therapies for the treatment of these infections, such as amikacin and imipenem, are expensive and difficult to acquire in Madagascar. Most ESBL-PE isolates are multidrug-resistant strains. Only 54.3% of isolates were susceptible to fluoroquinolones (FQ) on admission, and 47.8%, on discharge (Table 6). Previous FQ consumption has been proven to be a risk factor for acquisition of ESBL-producing strains, especially those producing CTX-M-type enzymes [29,31].

**Table 6:** Analysis of the Antibiotic therapies

ESBL-PE carriage		Univariate		Multivariate		
	OR	95%CI	p-value	OR adjusted	95%CI	p-value
Ampicillin	3.9	[1.6 - 9.9]	<0.01	3.5	[1.4 - 9.2]	<0.01
Gentamicin	2.4	[1.1 - 5.6]	0.04	2.0	[0.7 - 5.3]	0.15
Ceftriaxone	2.0	[0.8 - 4.4]	0.09	1.2	[0.5 - 3.2]	0.65

The significant increase in the prevalence of ESBL-PE carriage raises the concern of multidrug resistance in Madagascar. The presence of ESBL-PE complicates the selection of antibiotics used for the empirical therapy of community-onset infections. The only effective treatment for these strains (carbapenem and amikacin) (table 3) are not available in Madagascar, as they are too expensive. We fear that the increasing use of broad spectrum antibiotics may lead to an increased incidence of infections with ESBL-PE, whose treatment will be increasingly difficult. There is an urgent need to promote a rational use of antibiotics, both in hospital and in the community, the development of new generic drugs, and strict personal hygiene to prevent the selection and the spread of these strains.

## Conclusions

Nosocomial infections and antimicrobial resistance have become increasing challenges in low-income countries. The detection and quarantine of patients with ESBL-PE on admission to hospital are no longer adequate measures if a significant rate of carriage is highlighted in the community. Further studies are needed to quantify the prevalence of ESBL-PE carriage in the Malagasy population and to identify its determinants. The epidemic spread of resistance factors knows no borders. International collaboration is needed to help developing countries respond to the threat of infection with multidrug resistant bacteria.

This study was supported by a grant from the Institut Pasteur of Madagascar.

## Conflicts of interest

The authors declare that they have no competing interests.

## Authors' contributions

TA participated in the planning and execution of the study, performed data entry and data analysis, and was the principal investigator, FR performed the laboratory work. AT participated in the planning of the study, provided advices and participated in writing. HR was involved in planning the study. YB participated in data analysis and writing, and VR was the project coordinator and participated in planning, data analysis and writing. All authors read and approved the manuscript

## Note

Two First authors:

Dr Andriatahina is a physician. She had working in the Epidemiology Unit at the Institut Pasteur de Madagascar to validate MdH of IFMT (Laos).

Dr Randianirina has conducted biological research in Pasteur Institute of Madagascar for 5 years. She led the bacteriological analysis of this work.

These two authors contributed equally to this study

## Pre-publication history

The pre-publication history for this paper can be accessed here:

http://www.biomedcentral.com/1471-2334/10/204/prepub
